# BARRIERS AND FACILITATORS TO IMPLEMENTING THE BALANCING INCENTIVE PROGRAM (BIP)

**DOI:** 10.1093/geroni/igz038.559

**Published:** 2019-11-08

**Authors:** Lisa Beauregard, Edward A Miller

**Affiliations:** University of Massachusetts Boston, Boston, Massachusetts, United States

## Abstract

The Balancing Incentive Program (BIP) was an optional Medicaid program within the Affordable Care Act. States spending less than 50% of Medicaid long-term services and supports on home and community-based services (HCBS) were eligible for the program and could participate from 2011 to 2015. Participating states received an enhanced federal match in exchange for rebalancing LTSS spending and adopting structural changes to their long-term services and supports system. The purpose of this study is to understand the barriers and facilitators to implementing the BIP in two states. Data was collected through semi-structured interviews with individuals involved in HCBS policy nationally and in Maryland and Texas, including government bureaucrats, consumer advocates, and provider representatives. Findings indicate that factors that facilitated Maryland and Texas’ implementation of the BIP were regular communication with the Centers for Medicare and Medicaid Services and their consultants, Mission Analytics Group, merging the BIP with existing HCBS programs, and the substantial amount of funding associated with the program. On the other hand, the short duration of the BIP presented a challenge for states because they needed to enact multiple changes within a limited period of time. In addition, state procurement and contracting processes impeded the speed with which BIP requirements could be met. Key stakeholders, including consumer advocacy and provider organizations, often felt as though their state implemented the BIP with minimal input from interested groups. The findings indicate that the structure of the Balancing Incentive Program as well as internal state factors influenced the program’s implementation.

